# Draft Genome Sequence of “*Candidatus* Halobonum tyrrellensis” Strain G22, Isolated from the Hypersaline Waters of Lake Tyrrell, Australia

**DOI:** 10.1128/genomeA.01001-13

**Published:** 2013-12-12

**Authors:** Juan A. Ugalde, Priya Narasingarao, Sidney Kuo, Sheila Podell, Eric E. Allen

**Affiliations:** Marine Biology Research Division, Scripps Institution of Oceanography, University of California, San Diego, La Jolla, California, USAa; Division of Biological Sciences, University of California, San Diego, La Jolla, California, USAb

## Abstract

We report the draft 3.675-Mbp genome sequence of “*Candidatus* Halobonum tyrrellensis” strain G22, a novel halophilic archaeon isolated from the surface hypersaline waters of Lake Tyrrell, Australia. The availability of the first genome from the “*Candidatus* Halobonum” genus provides a new genomic resource for the comparative genomic analysis of halophilic *Archaea*.

## GENOME ANNOUNCEMENT

Halophilic *Archaea* of the class *Halobacteria* (phylum *Euryarchaeota*) are dominant members of extreme hypersaline environments worldwide (1). Numerous genera have been isolated from diverse hypersaline habitats, and many representative genome sequences are available (1, 2). However, recent metagenomic analyses of hypersaline ecosystems have revealed that these reference halophiles are not adequately representative of the dominant microbial populations present in many natural hypersaline habitats (3–5). Here, we report the genome sequence of a novel member of the class *Halobacteria* isolated from the hypersaline surface waters of Lake Tyrrell, Victoria, Australia.

Surface water samples were plated onto minimal medium containing a 23% salt solution amended with various carbon substrates, including glycerol, acetate, or glucose, and incubated at room temperature under aerobic conditions. After 3 to 4 weeks of incubation, the colonies were restreaked for purity and characterized via 16S rRNA gene amplification and sequencing to screen for novel species/strains. “*Candidatus* Halobonum tyrrellensis” strain G22 was isolated from minimal medium containing glycerol as the sole carbon source, incubated aerobically at room temperature. Genomic DNA was sequenced using 454 Titanium chemistry at the J. Craig Venter Institute (Rockville, MD). The total number of reads generated was 568,949, with an average length of 428 bp. The sequences were assembled using Newbler (version 2.7), resulting in a total of 72 contigs (N_50_, 119,067 bp; mean contig length, 45,962 bp; maximum contig length, 303,316 bp), with an estimated genome size of 3,675,087 bp and a G+C content of 70.1%. Functional annotation of predicted gene sequences was performed using the IMG-ER platform (6). A total of 3,525 predicted coding sequences were identified, including 47 tRNAs and a single copy of the rRNA operon.

A phylogenetic tree based on 16S rRNA genes (http://dx.doi.org/10.6084/m9.figshare.830514) suggests that “*Ca*. Halobonum tyrrellensis” is a member of the *Halobacteriaceae* family and a sister group of the *Halobaculum* genus, sharing 92% 16S rRNA gene sequence identity with *Halobaculum gomorrense* (7). A comparison of the “*Ca*. Halobonum tyrrellensis” genome with the partial genome sequence available for *H. gomorrense* (632,433 bp) (8) revealed an average nucleotide identity (ANI) of 81.56 ± 1.18%. A phylogenomic approach using multiple amino acid markers (9) supports the placement of “*Ca*. Halobonum tyrrellensis” as a new genus (http://dx.doi.org/10.6084/m9.figshare.830514). A detailed characterization of the physiology and metabolism of “*Ca*. Halobonum tyrrellensis” and a formal description of this strain are currently in progress.

The features found in the genome include the presence of a putative sensory rhodopsin, a high number of ABC transporters and carbon metabolism genes, including trehalose utilization genes, and the absence of conserved haloarchaeal genes encoding a flagellar system or gas vesicle synthesis proteins.

The “*Ca*. Halobonum tyrrellensis” genome represents the first high-quality draft sequence for a member of the new candidate genus *Halobonum*. These data expand the breadth of the reference genome sequence information for halophilic *Archaea*, providing a new resource for comparative genomic analyses and the phylogenetic binning of metagenomic sequence data recovered from hypersaline environments.

### Nucleotide sequence accession number.

The draft genome sequence of “*Ca.* Halobonum tyrrellensis” strain G22 is deposited at DDBJ/EMBL/Genbank databases under the accession no. ASGZ00000000.
